# Mortality in older adults following a fragility fracture: real-world retrospective matched-cohort study in Ontario

**DOI:** 10.1186/s12891-021-03960-z

**Published:** 2021-01-23

**Authors:** Jacques P. Brown, Jonathan D. Adachi, Emil Schemitsch, Jean-Eric Tarride, Vivien Brown, Alan Bell, Maureen Reiner, Thiago Oliveira, Ponda Motsepe-Ditshego, Natasha Burke, Lubomira Slatkovska

**Affiliations:** 1 CHU de Québec Research Centre and Laval University, Québec, QC Canada; 2 Department of Medicine, McMaster University, Hamilton, ON Canada; 3 Division of Orthopaedic Surgery, Western University, London, ON Canada; 4 Department of Health Research Methods, Evidence and Impact (HEI), McMaster University, Hamilton, ON Canada; 5 Programs for Assessment of Technology in Health, The Research Institute of St. Joe’s Hamilton, St Joseph’s Healthcare Hamilton, Hamilton, ON Canada; 6 Centre for Health Economics and Policy Analysis (CHEPA), McMaster University, Hamilton, ON Canada; 7 Department of Family and Community Medicine, University of Toronto, Toronto, Canada; 8 Amgen Inc, California, USA; 9 Amgen Canada Inc., Toronto, Canada

**Keywords:** Fracture, Osteoporosis, Mortality, Real-world, Older adults

## Abstract

**Background:**

Recent studies are lacking reports on mortality after non-hip fractures in adults aged > 65.

**Methods:**

This retrospective, matched-cohort study used de-identified health services data from the publicly funded healthcare system in Ontario, Canada, contained in the ICES Data Repository. Patients aged 66 years and older with an index fragility fracture occurring at any osteoporotic site between 2011 and 2015 were identified from acute hospital admissions, emergency and ambulatory care using International Classification of Diseases (ICD)-10 codes and data were analyzed until 2017. Thus, follow-up ranged from 2 years to 6 years. Patients were excluded if they presented with an index fracture occurring at a non-osteoporotic fracture site, their index fracture was associated with a trauma code, or they experienced a previous fracture within 5 years prior to their index fracture. This fracture cohort was matched 1:1 to controls within a non-fracture cohort by date, sex, age, geography and comorbidities. All-cause mortality risk was assessed.

**Results:**

The survival probability for up to 6 years post-fracture was significantly reduced for the fracture cohort vs matched non-fracture controls (*p* < 0.0001; *n* = 101,773 per cohort), with the sharpest decline occurring within the first-year post-fracture. Crude relative risk of mortality (95% confidence interval) within 1-year post-fracture was 2.47 (2.38–2.56) in women and 3.22 (3.06–3.40) in men. In the fracture vs non-fracture cohort, the absolute mortality risk within one year after a fragility fracture occurring at any site was 12.5% vs 5.1% in women and 19.5% vs 6.0% in men. The absolute mortality risk within one year after a fragility fracture occurring at a non-hip vs hip site was 9.4% vs 21.5% in women and 14.4% vs 32.3% in men.

**Conclusions:**

In this real-world cohort aged > 65 years, a fragility fracture occurring at any site was associated with reduced survival for up to 6 years post-fracture. The greatest reduction in survival occurred within the first-year post-fracture, where mortality risk more than doubled and deaths were observed in 1 in 11 women and 1 in 7 men following a non-hip fracture and in 1 in 5 women and 1 in 3 men following a hip fracture.

**Supplementary Information:**

The online version contains supplementary material available at 10.1186/s12891-021-03960-z.

## Background

The link between osteoporosis-related fragility fractures and mortality has been established by studies conducted across the globe over the last 3 decades, especially for hip fractures [1–10]. These studies show that in different countries with different types of healthcare systems, a fragility fracture was associated with a greater risk of mortality within the first year following the fracture and remained increased for several years after. Similar results have been reported in Canada [4, 7–10], whereby a fragility fracture occurring at any osteoporotic site increased mortality risk by 1.4–1.5-fold in adults aged 50 years and older [4]. When examining specific fragility fracture sites, a 2–4-fold mortality risk increase was observed after hip, vertebral or humerus (in men) fractures in Canadians aged 50 and older [4, 7, 8], while several other fracture sites were found to be associated with a 1.4–2-fold mortality risk increase (rib, humerus, forearm, or pelvic fracture in women; wrist fracture in men) [4, 8].

In spite of this mortality burden, a large gap currently exists for the primary prevention of fracture, and studies show that an estimated 80–90% of adults do not receive appropriate osteoporosis management even in the secondary prevention setting [11–13]. In contrast, approximately 90% of patients with cardiovascular disease are provided appropriate secondary preventive care [14]. System-level and country-level healthcare initiatives have been implemented worldwide to focus on addressing the secondary fracture prevention gap [15, 16], with several reports showing these initiatives being effective in not only reducing subsequent fragility fracture rates but also associated mortality [17–20]. Considering these data and the current fracture prevention gap, it is important to further improve knowledge of mortality associated with fragility fractures among medical or non-medical professionals involved in osteoporosis care [11–13, 21–23].

Studies examining post-fracture mortality after non-hip fractures over the past decade have typically focused on younger patient populations (aged ≥ 50 years), while studies examining mortality risk in adults aged > 65 have focused primarily on hip fractures [4, 7–10]. As such, there is a gap in understanding the mortality risks post fracture occurring at any or non-hip osteoporotic sites in adults aged > 65. Within this group, due to age-related fracture risk increase, more serious recommendations currently exist for fracture risk assessment or pharmacological intervention for osteoporosis [24, 25], and therefore the closing of the fracture prevention gap is that much more important.

The primary objective of this study was to utilize health services data from the publicly funded healthcare system in Canada to assess mortality post-fracture in a large, real-world cohort of adults aged > 65 years who experienced an index fragility fracture of any osteoporotic and non-hip site between 2011 and 2015, in comparison to matched non-fracture controls. Secondarily, surgeries and complications, which partly relate to mortality, were analyzed for all fragility fracture sites examined in this study.

## Methods

### Study design

A population-based retrospective matched-cohort study was conducted using public healthcare databases including health services records for about 13 million people living in Ontario, Canada. Primary databases used to identify and describe cases in this study were the Registered Persons Database (RPDB), Discharge Abstract Database (DAD)/ Same Day Surgery (SDS), National Ambulatory Care Reporting System (NACRS), and Ontario Health Insurance Plan (OHIP) (Supplementary Table 1). Healthcare encounters were recorded in multiple record-level, administrative datasets in the ICES Data Repository, and encrypted patient-specific identifiers (ICES-specific key number [IKN]) were used to link the administrative datasets.

### Participants and setting

Included in the fracture cohort were Ontario residents aged 66 years and older (> 65) with an index fracture occurring at an osteoporotic fracture site from January 1, 2011 to March 31, 2015, identified from acute hospital admissions, emergency and ambulatory care using International Classification of Diseases (ICD)-10 diagnostic codes for fracture (Supplementary Table 2) as a main diagnosis or admitting diagnosis (ie, if no definite diagnosis was made, the main symptom, abnormal finding or problem was selected) [26]. Patients with fractures occurring at the following osteoporotic fracture sites were included in the fracture cohort: hip, vertebral (clinical), wrist (distal radius, or both distal radius and ulna), clavicle/sternum, rib, humerus, tibia/fibula/knee (including medial and lateral malleolus), pelvis, radius/ulna (proximal, midshaft, or distal ulna only), multisite, femur. Patients were excluded if they presented with an index fracture of non-osteoporotic fracture sites (ie, skull, face, hands, and feet) or their index fracture was associated with a trauma code (Supplementary Table 3) to maximize the probability that only fragility fractures were examined [9]. Patients were also excluded if they experienced a previous fracture during the 5-year lookback period prior to the index fracture date to minimize the likelihood that post index fracture mortality was influenced by a recent fracture occurring prior to an index event.

Patients in the fracture cohort were matched 1:1 to controls (non-fracture cohort) from the Registered Persons Database (RPDB) who did not experience a fracture between January 1, 2011 and March 31, 2015 or during the 5-year lookback period prior to their index date. Matching was performed based on the following a priori specified variables: month and year of index date (random index date assigned to controls); sex; age groups (66–70, 71–75, 76–80, 81–85, ≥86 years); geography (rural/urban); and comorbidities (respiratory conditions: asthma, chronic obstructive pulmonary disorder [COPD]; inflammatory conditions: rheumatoid arthritis, psoriasis, spondylarthritis; cancer within 5 years; chronic kidney disease; diabetes; vascular events: myocardial infarction, stroke and cerebrovascular events; dementia; and osteoarthritis).

Data were analysed from the index date until March 31, 2017 (Supplementary Fig. 1). Index date was defined as the date of the index fracture in the fracture cohort and the date of when the follow-up was initiated in the non-fracture cohort. Thus, depending on the index date, the opportunity for follow-up ranged between 2 years (2015–2017) and 6 years (2011–2017). Data from 5 years prior to the index date were also collected to describe clinical characteristics in both cohorts.

### Study size

The fracture cohort included all Ontario residents who experienced a fragility fracture that were registered in the DAD/SDS and NACRS using the above-specified criteria for osteoporotic fracture sites and non-trauma fractures, and were able to be matched to the non-fracture controls using the above-specified matching criteria (see Participants and setting section). Both sets of criteria were specified a priori to minimize selection bias. The cohort was limited to those aged 66 years and older (> 65) to collect medication data based on public drug coverage for at least 1 year prior to the index fracture. The 2011–2015 index period was selected to allow at least 2 years of follow-up post-index date and was based on data availability in the ICES Data Repository when the data collection started in 2018.

### Variables and data sources

#### Identifying mortality

Deaths occurring due to any cause from the index date until end of the study period were obtained from the RPDB for both fracture and non-fracture cohorts.

#### Identifying fracture-related surgeries and complications

Fracture-related orthopedic surgical procedures (initial and revision) were assessed from the date of index fracture until the end of follow-up in the fracture cohort using the DAD/SDS and Canadian Classification of Health Interventions (CCI) codes (Supplementary Table 4), and surgery-related complications were assessed up to 30 days after an index event using the DAD / SDS, NACRS, and OHIP databases based on ICD-9 or 10 codes (Supplementary Table 5). Vertebroplasty and balloon kyphoplasty were not included in the analysis of fracture-related orthopedic surgical procedures, because these procedures are typically not performed as part of the standard initial orthopedic management of vertebral fractures, as their primary aim is to minimize complications associated with severe and/or multiple vertebral fractures (kyphosis). For deaths and surgical procedures, a minimum follow-up of 1 year was required, but longer follow-up was permitted.

#### Identifying fractures

Index and second fragility fractures occurring during the follow-up period were examined in the fracture cohort using the DAD/SDS for inpatient visits and NACRS for emergency and ambulatory visits. For the second fragility fractures, the same criteria were applied to examine fractures occurring at osteoporotic sites without trauma as for the index fractures (see Participants and setting section). Fracture of the same site that was dated within 91 days of the index fracture was assumed to stem from the same fracture and was not counted as a second fracture [27]. The anatomical location of multisite index fracture was used to exclude a subsequent single-site fracture occurring in a similar location within 91 days.

#### Identifying clinical characteristics

The following databases were used to assess clinical characteristics at the time of the index date in the fracture and non-fracture cohort: RPDB for age and sex; Ontario Drug Benefit (ODB) for medications; and DAD/SDS, NACRS, OHIP, Ontario Rheumatoid Arthritis Administrative Database, Ontario Cancer Registry, Ontario Renal Reporting System, Ontario Diabetes Database, Ontario Myocardial Infarction Database, ASTHMA, COPD for comorbidities using the ICD-9 or ICD-10 codes where applicable.

### Statistical methods

Cumulative proportions of deaths due to any cause within 1, 2 or 3 years after an index fracture occurring at any site (fracture cohort) or start of follow-up (non-fracture cohort) were calculated separately for women and men and expressed as absolute risks and absolute risk differences (fracture cohort – non-fracture cohort). Crude relative risks were calculated for women and men (fracture cohort/non-fracture cohort). Adjustment for confounders was not deemed necessary in light of these large cohorts being matched on many a priori specified potential confounders. Survival analysis was conducted using Kaplan-Meier estimates with log rank statistics for evaluation of statistical significance for comparisons between the fracture and non-fracture cohorts.

Cumulative proportions of deaths due to any cause within 1, 2 or 3 years after index fracture were further stratified for fracture cohort participants by hip vs non-hip fracture site and by age categories (66–70, 71–75, 76–80, 81–85, 86+ years). For each fracture site, proportions of deaths, fracture-related surgeries or complications within 1 year were calculated.

## Results

### Clinical characteristics

**Fig. 1:**
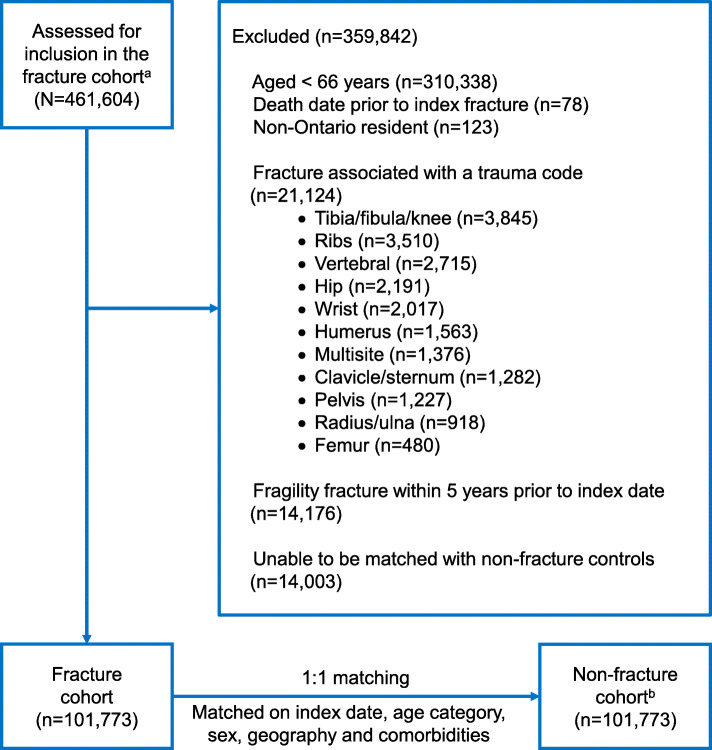
Flow diagram of fracture and non-fracture cohort adults included in the study. Note: ICD-10-CA, International Classification of Diseases, 10th revision, Canada; IKN, ICES key number. ^a^All patients with valid IKN with a non-trauma fracture occurring at an osteoporotic fracture site (hip, humerus, vertebral, wrist, pelvis, femur, clavicle/sternum, ribs, radius/ulna, or tibia/ fibula/knee) between January 1, 2011 and March 31, 2015. Fractures were identified using ICD-10-CA codes from hospital admissions, emergency room visits, and ambulatory care. ^b^Controls who did not experience a fracture between January 1, 2011 and March 31, 2015 or during the 5-year lookback period prior to their index date

A total of 115,776 eligible adults (72.3% female) > 65 years sustained an index fragility fracture between January 1, 2011 and March 31, 2015. From the full, unmatched cohort of 115,776 adults with fragility fracture a subset of 101,773 (88%) were able to be matched 1:1 with non-fracture controls based on a priori specified variables including index date, age category, sex, geography and comorbidities (Fig. 1). All clinical characteristics used for matching were similar between the matched fracture and non-fracture cohorts (Table 1). Mean age (*p* = 0.04) and osteoporotic medication use 1 year prior to the index date (any, bisphosphonates, denosumab, or raloxifene; *p* < 0.001) were statistically different between the matched cohorts (Table 1); these two characteristics were however not used for matching and the difference in each was small and deemed not clinically relevant. The full unmatched and matched fracture cohorts had similar clinical characteristics and proportions of different index and subsequent fractures sites (Table 1; Supplementary Table 6).

**Table 1:** Clinical characteristics of the fracture cohort and non-fracture cohorts

Clinical Characteristics	Fracture cohort n (%)	Non-fracture cohort n (%)
**Total number of patients**	101,773	101,773
**Sex**		
Female	74,557 (73.3%)	74,557 (73.3%)
Male	27,216 (26.7%)	27,216 (26.7%)
**Age**		
Mean ± SD^a^	80.25 ± 8.37*	80.33 ± 8.75*
Median (IQR)^a^	81 (73–87)	81 (73–87)
66–70 years	16,672 (16.4%)	16,672 (16.4%)
71–75 years	15,996 (15.7%)	15,996 (15.7%)
76–80 years	17,952 (17.6%)	17,952 (17.6%)
81–85 years	20,584 (20.2%)	20,584 (20.2%)
≥86 years	30,569 (30.0%)	30,569 (30.0%)
**Urban Residence**	89,696 (88.1%)	89,696 (88.1%)
**Respiratory conditions**^**b**^		
Asthma	13,113 (12.9%)	13,113 (12.9%)
COPD	25,991 (25.5%)	25,991 (25.5%)
**Inflammatory conditions**^**b**^		
Rheumatoid arthritis	2208 (2.2%)	2208 (2.2%)
Psoriasis	4985 (4.9%)	4985 (4.9%)
Spondyloarthritis	2432 (2.4%)	2432 (2.4%)
**Cancer**^**b**^	5166 (5.1%)	5166 (5.1%)
**Chronic kidney disease**^**b**^	8909 (8.8%)	8909 (8.8%)
**Diabetes**^**b**^	29,074 (28.6%)	29,074 (28.6%)
**Vascular events**^**b**^		
Myocardial infarction	4549 (4.5%)	4549 (4.5%)
Stroke or cerebrovascular events	28,015 (27.5%)	28,015 (27.5%)
**Osteoarthritis**^**b**^	77,526 (76.2%)	77,526 (76.2%)
**Dementia**^**b**^	18,359 (18.0%)	18,359 (18.0%)
**Osteoporosis treatment type within 1 year prior**^**a,c**^		
Any treatment	28,974 (28.5%)**	21,179 (20.8%)**
Denosumab	1383 (1.4%)**	1088 (1.1%)**
Bisphosphonate	25,626 (25.2%)**	17,720 (17.4%)**
Raloxifene	599 (0.6%)**	465 (0.5%)**
HRT	3259 (3.2%)	3312 (3.3%)
**Index fracture by site**^**a,d**^		
Hip	26,963 (26.5%)	–
Wrist	16,467 (16.2%)	–
Humerus	11,756 (11.6%)	–
Ribs	10,247 (10.1%)	–
Tibia/fibula/knee	9859 (9.7%)	–
Pelvis	7209 (7.1%)	–
Vertebral	6595 (6.5%)	–
Radius/ulna	4377 (4.3%)	–
Multisite	3299 (3.2%)	–
Femur	2618 (2.6%)	–
Clavicle/sternum	2383 (2.3%)	
Any site	101,773 (100%)	
**Second fracture by site**^**a,d**^		
Hip	4956 (4.9%)	–
Wrist	2002 (2.0%)	–
Humerus	1814 (1.8%)	–
Pelvis	1722 (1.7%)	–
Ribs	1647 (1.6%)	–
Vertebral	1590 (1.6%)	–
Multisite	1326 (1.3%)	–
Tibia/fibula/knee	1164 (1.1%)	–
Radius/ulna	650 (0.6%)	–
Femur	620 (0.6%)	–
Clavicle/sternum	467 (0.5%)	
Any site	17,958 (17.6%)	

Values reported as n (%) unless otherwise indicated; percent of total respective cohort

*p < 0.05, **p < 0.001 statistical significance between fracture cohort and non-fracture cohort

^a^Variable not used for matching

^b^Time frame for cancer was 5 years within index data and for all other comorbidities any time prior to index date

^c^Within 1 year of index date. Bisphosphonates include alendronate, cyclical etidronate, risedronate, or zoledronic acid. Denosumab is not publicly covered in men and teriparatide in men or women in Ontario

^d^Index fragility fracture cases from January 1, 2011 to March 31, 2015. Second fragility fracture cases from the date of index event to March 31, 2017. Reported from highest to lowest number

Within the fracture cohort, fractures occurring at the hip, wrist, humerus, and rib were the four most common fractures, representing approximately two thirds of index or second fracture sites (Table 1). Index hip fractures alone represented 26.5% of index fractures (*n* = 26,963). By the end of follow-up (maximum 6 years), a second fracture of any site occurred in 17.6% (*n* = 17,958) of the fracture cohort, while a second hip fracture occurred in 4.9% (*n* = 4956). The median length of study follow-up was 3.26 years or 1190 days (Supplementary Table 7).

### Survival and mortality after index fragility fracture with all sites combined

**Fig. 2:**
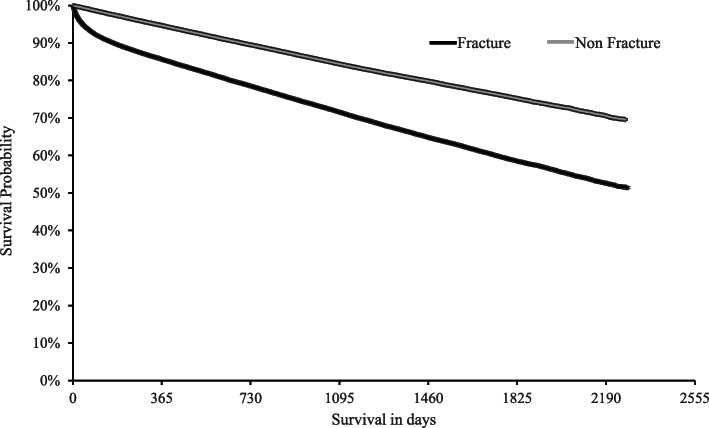
Survival probability in the fracture cohort and non-fracture cohort. Note: Thickness of fracture and no fracture cohort curves represent 95% confidence intervals

Survival analyses demonstrated a statistically significant difference in survival probability (*p* < 0.0001) between the fracture and non-fracture cohort over 6 years of follow-up (Fig. 2). Visual inspection of the Kaplan-Meier curve for the fracture cohort revealed a sharp initial drop in the proportion of individuals alive within 1-year post-index fracture. The curve continued to further separate from the non-fracture cohort over the remaining 5 years of follow-up, albeit not as steeply. Survival probability (95% CI) in the fracture vs non-fracture cohorts was 85.6% (85.4–85.9%) vs 94.7% (94.5–94.8%) within 1-year post-fracture and 58.5% (58.1–58.9%) vs 75.2% (74.9–75.6%) within 5 years post-fracture. The following cumulative proportions of patients died over the three year post-fracture period in the fracture-cohort and the non-fracture cohort: 14.4% (*n* = 14,617) and 5.3% (*n* = 5418) in year 1; 21.5% (*n* = 21,854) and 10.5% (*n* = 10,676) in year 2, and 28.4% (*n* = 28,952) and 15.6% (*n* = 15,849) in year 3.

The absolute risk of death was significantly greater in women and men in the fracture cohort vs non-fracture cohort (Table 2). At 1, 2 and 3 years post index date, the absolute risk difference was, respectively, 7.4% (95% CI 7.1–7.7%), 9.1% (95% CI 8.7–9.4%) and 10.8% (95% CI 10.4–11.2%) in women and 13.5% (95% CI 12.9–14.0%), 16.2% (95% CI 15.5–16.8%) and 18.5% (95% CI 17.7–19.2%) in men. Within 1, 2 and 3 years post-index date, the unadjusted relative risk was, respectively, 2.47 (95% CI 2.38–2.56), 1.92 (95% CI 1.87–1.97), and 1.73 (95% CI 1.70–1.77) in women and 3.22 (95% CI 3.06–3.40), 2.34 (95% CI 2.26–2.43) and 2.04 (95% CI 1.98–2.10) in men. The absolute risk of death was 1.4–1.6x higher in men vs women with fractures over the three years post-fracture, and 1.2x higher in men vs women without fractures.

**Table 2:** Absolute risk of death and number of deaths in the fracture cohort and the non-fracture cohort

Mortality	Fracture cohort				Non-fracture cohort			
	Female (***N*** = 74,557)		Male (***N*** = 27,216)		Female (***N*** = 74,557)		Male (***N*** = 27,216)	
	Number of deaths	% deaths (95% CI)^**a**^	Number of deaths	% deaths (95% CI)^**a**^	Number of deaths	% deaths (95% CI)^a^	Number of deaths	% deaths (95% CI)^**a**^
Within 1 year								
*All ages*	9306	12.5 (12.2, 12.7)	5311	19.5 (19.0, 20.0)	3772	5.1 (4.9, 5.2)	1646	6.0 (5.8, 6.3)
*66–70 years*	410	3.5 (3.2, 3.8)	340	6.9 (6.2, 7.7)	115	1.0 (0.8, 1.2)	70	1.4 (1.1, 1.8)
*71–75 years*	588	5.2 (4.8, 5.6)	501	10.7 (9.8, 11.7)	188	1.7 (1.4, 1.9)	134	2.9 (2.4, 3.4)
*76–80 years*	1007	7.8 (7.3, 8.3)	835	16.5 (15.4, 17.7)	379	2.9 (2.7, 3.3)	214	4.2 (3.7, 4.8)
*81–85 years*	1739	11.6 (11.1, 12.2)	1226	21.9 (20.7, 23.1)	722	4.8 (4.5, 5.2)	409	7.3 (6.6, 8.0)
*86+ years*	5562	23.6 (22.9, 24.2)	2409	34.6 (33.3, 36.0)	2368	10.0 (9.6, 10.4)	819	11.8 (11.0, 12.6)
*Hip index fracture (all ages)*	4126	21.5 (20.8, 22.1)	2499	32.3 (31.1, 33.6)	.		.	
*Non-hip index fracture (all ages)*	5180	9.4 (9.1, 9.6)	2812	14.4 (13.9, 15.0)	.		.	
Within 2 years								
*All ages*	14,171	19.0 (18.7, 19.3)	7683	28.2 (27.6, 28.9)	7396	9.9 (9.7, 10.1)	3280	12.1 (11.6, 12.5)
*66–70 years*	628	5.3 (4.9, 5.8)	539	10.9 (10.0, 11.9)	241	2.1 (1.8, 2.3)	151	3.1 (2.6, 3.6)
*71–75 years*	936	8.3 (7.7, 8.8)	757	16.2 (15.1, 17.4)	376	3.3 (3.0, 3.7)	264	5.6 (5.0, 6.4)
*76–80 years*	1605	12.4 (11.8, 13.1)	1207	23.9 (22.5, 25.2)	765	5.9 (5.5, 6.4)	458	9.1 (8.2, 9.9)
*81–85 years*	2771	18.5 (17.8, 19.2)	1801	32.1 (30.7, 33.7)	1478	9.9 (9.4, 10.4)	814	14.5 (13.5, 15.6)
*86+ years*	8231	34.9 (34.1, 35.6)	3379	48.6 (47.0, 50.3)	4536	19.2 (18.7, 19.8)	1593	22.9 (21.8, 24.1)
*Hip index fracture (all ages)*	5784	30.1 (29.3, 30.9)	3322	43.0 (41.5, 44.4)	.		.	
*Non-hip index fracture (all ages)*	8387	15.2 (14.8, 15.5)	4361	22.4 (21.7, 23.1)	.		.	
Within 3 years								
*All ages*	19,084	25.6 (25.2, 26.0)	9868	36.3 (35.5, 37.0)	11,010	14.8 (14.5, 15.0)	4839	17.8 (17.3, 18.3)
*66–70 years*	852	7.3 (6.8, 7.8)	716	14.5 (13.5, 15.6)	390	3.3 (3.0, 3.7)	258	5.2 (4.6, 5.9)
*71–75 years*	1290	11.4 (10.8, 12.0)	989	21.2 (19.9, 22.5)	576	5.1 (4.7, 5.5)	402	8.6 (7.8, 9.5)
*76–80 years*	2276	17.7 (16.9, 18.4)	1605	31.7 (30.2, 33.3)	1214	9.4 (8.9, 10.0)	693	13.7 (12.7, 14.8)
*81–85 years*	3862	25.8 (25.0, 26.6)	2396	42.7 (41.1, 44.5)	2291	15.3 (14.7, 15.9)	1230	21.9 (20.7, 23.2)
*86+ years*	10,804	45.8 (44.9, 46.6)	4162	59.8 (58.0, 61.7)	6539	27.7 (27.0, 28.4)	2256	32.4 (31.1, 33.8)
*Hip index fracture (all ages)*	7495	39.0 (38.1, 39.9)	4094	52.9 (51.3, 54.6)	.		.	
*Non-hip index fracture (all ages)*	11,589	20.9 (20.6, 21.3)	5774	29.6 (28.9, 30.4)	.		.	

^**a**^Percent of the total respective subgroup by age category or hip / non-hip fracture site

### Mortality after index fractures stratified by age or fracture site

In women, the range for the absolute risk of death observed between the youngest (66–70) and oldest (86+) age groups at year 1, 2, and 3 was, respectively, 3.5–23.6%, 5.3–34.9%, and 7.3–45.8% (Table 2), representing 6.3–6.7-fold difference between the youngest and oldest groups. The absolute risk of death in women with vs without fractures aged 66–70, 71–75, 76–80, 81–85, 86+ years, respectively, was higher by 3.6-, 3.1-, 2.7-, 2.4-, and 2.3-fold at year 1; 2.6-, 2.5–2.1-, 1.9-, and 1.8-fold at year 2; and 2.2-, 2.2-, 1.9-, 1.7-, and 1.7-fold at year 3. In men, the range for the absolute risk of death observed between the youngest (66–70) and oldest (86+) age group at year 1, 2, and 3 was, respectively, 6.9–34.6%, 10.9–48.6%, and 14.5–59.8%, representing 4.1–5.0-fold difference between the youngest and oldest groups. The absolute risk of death in men with vs without fractures aged 66–70, 71–75, 76–80, 81–85, 86+ years, respectively, was higher by 4.9-, 3.7-, 3.9-, 3.0-, and 2.9-fold at year 1; 3.6-, 2.9-, 2.6-, 2.2-, and 2.1-fold at year 2; and 2.8-, 2.5-, 2.3-, 1.9-, and 1.8-fold at year 3. Compared to women with fractures, the absolute risk of death over the three years post-fracture was 1.7–2.1x higher in men aged 66–85 years. This relative difference between sexes declined in the oldest, 86+ age group and was 1.3–1.5x higher in men vs women. This age-sex trend was also present in the non-facture cohort, with 1.4–1.7x higher mortality rate in men vs women aged 66–85 years and 1.2x in those aged 86+ over the three years post-index date.

The absolute risk of death in women (all ages) with hip fracture, with non-hip fracture and without fracture was, respectively, 21.5–39.0%, 9.4–20.9% and 5.1–14.8% over the three years post-index date, and 32.3–52.9%, 14.4–29.6% and 6.0–17.8% in men (all ages) (Table 2). The absolute risk of death in women 1) with hip fractures vs without fractures, 2) with non-hip fractures vs without fractures, and 3) with hip vs non-hip fracture, respectively, was higher by 4.2-, 1.9-, and 2.3-fold at year 1, 3.0-, 1.5-, and 2.0-fold at year 2, and 2.6-, 1.4-, and 1.9-fold at year 3. The absolute risk of death in men 1) with hip fractures vs without fractures, 2) with non-hip fractures vs without fractures, and 3) with hip vs non-hip, respectively, was higher by 5.3, 2.4-, and 2.2-fold at year 1, 3.6-, 1.9- and 1.9-fold at year 2, and 3.0-, 1.7- and 1.8-fold at year 3. The absolute risk of death was 1.4–1.5x higher in men vs women with hip fractures over the three years post-fracture, and it was also 1.4–1.5x higher in men vs women with non-hip fractures. In terms of the contribution to the total number of mortality cases in the fracture cohort, hip fractures contributed 45.3% (*n* = 6625) of the total number of deaths within one year post-fracture.

Compared to 1-year absolute risk of death observed in the non-fracture cohort (5.3%), wrist (4.4%), radius/ulna (6.0%), and tibia/fibula/knee (5.8%) index fracture cases had similar mortality rates (Table 3). Femur (20.2%), multisite (18.4%), pelvis (18.0%) and vertebral (17.9%) index fractures were associated with the highest 1-year mortality rate following hip fracture (24.6%). In terms of the highest contribution to the total number of mortality cases, hip, rib, pelvis, humerus and vertebral index fractures together contributed 79.3% (*n* = 11,592) of the total number of deaths within one year post-fracture.

**Table 3:** Subsequent outcomes based on index fracture site in the fracture cohort

Index Fracture type	Number of index fractures (%)	Initial index fracture-related surgical procedure n (%)	Revision index fracture-related surgical procedure n (%) ^a^	Complications (at 30 days) n (%)^**b**^	Mortality at 1-year post-index fracture n (%)
Hip	26,963 (26.5%)	24,635 (91.4%)	1311 (5.3%)	5295 (21.5%)	6625 (24.6%)
Wrist	16,467 (16.2%)	3177 (19.3%)	237 (7.5%)	205 (6.5%)	718 (4.4%)
Humerus	11,756 (11.6%)	2444 (20.8%)	269 (11.0%)	285 (11.7%)	1159 (9.9%)
Ribs	10,247 (10.1%)	641 (6.3%)	84 (13.1%)	64 (10.0%)	1327 (13.0%)
Tibia, fibula, knee	9859 (9.7%)	2747 (27.9%)	437 (15.9%)	356 (13.0%)	575 (5.8%)
Pelvis	7209 (7.1%)	638 (8.9%)	77 (12.1%)	75 (11.8%)	1298 (18.0%)
Vertebral	6595 (6.5%)	573 (8.7%)	85 (14.8%)	81 (14.1%)	1183 (17.9%)
Radius and ulna	4377 (4.3%)	510 (11.7%)	40 (7.8%)	47 (9.2%)	264 (6.0%)
Multisite	3299 (3.2%)	1423 (43.1%)	121 (8.5%)	276 (19.4%)	608 (18.4%)
Femur	2618 (2.6%)	2124 (81.1%)	355 (16.7%)	736 (34.7%)	530 (20.2%)
Clavicle/sternum	2383 (2.3%)	228 (9.6%)	38 (16.7%)	26 (11.4%)	325 (13.6%)
**Total (all fracture types)**	**101,773 (100%)**	**39,140 (38.5%)**	**3054 (7.8%)**	**7446 (19.0%)**	**14,612 (14.4%)**

^**a**^Of those that had an initial replacement procedure

^**b**^Of those that had an initial replacement procedure; each complication was checked up to 30 days after the index fracture; complications included: infections related to surgery, venous thromboembolism (DVT and PE), pneumonia, myocardial infarction, complications related to prosthetic devices, refracture due to the surgical procedure

### Surgeries and complications by index fracture site

Within the fracture cohort, 38.5% of adults required an index fracture-related surgical procedure, of which 7.8% had revision surgeries, and 19.0% experienced post-surgical complications (Table 3). Index fracture sites of the hip, femur and multisite were associated with the highest proportion of fracture cohort patients undergoing initial surgical procedures (91.4, 81.1 and 43.1%), experiencing complications (21.5, 34.7 and 19.4%), as well as dying within 1-year post-fracture (24.6, 20.2 and 18.4%).

## Discussion

This study of adults aged > 65 confirms that a fragility fracture of any site is associated with increased mortality risk [4]. Survival declined over 1–6 years post-fracture with the steepest reduction occurring within the first year, when the crude relative risk was 2.47- and 3.22-fold higher in matched fractured vs non-fractured women and men, respectively. Absolute mortality risk within 1-year post-fracture was 12.5% in women with fractures and 19.5% in men with fractures, with a large absolute risk difference when compared to matched non-fracture controls of 7.4% (95% CI 7.1–7.7%) in women and 13.5% (95% CI 12.9–14.0%) in men.

To add context to the 1-year absolute mortality risk of 14.4% post-index fracture at any site observed in our fracture cohort of women and men (50% aged ≥81), a 1-year absolute mortality risk of 9.7% was observed post-index acute myocardial infarction in Canadians admitted to a hospital between 2006 and 2010 (22% aged 65–74, 35% aged ≥75) [28]. When considering stroke-related mortality, an absolute mortality risk of 19.3% was reported at discharge in Canadians hospitalised due to ischemic stroke between 2003 and 2004 (aged ≥70, 55% aged > 80) [29]. Furthermore, an absolute mortality risk of 14.9% was observed within 30 days in-hospital in Canadians admitted to hospital with any stroke in 2012 (25% aged 70–79, 38% aged ≥80) [30]. Considering that significant reductions in mortality risks associated with cardiovascular events have been reported over the past few decades [30–32], this data demonstrating a clear association between fragility fracture and mortality risk further highlights the need for effective measures to improve osteoporosis management and prevention of fragility fractures in older adults [11–13, 17–19, 21–23].

Only one study published over the last decade examined mortality post-fragility fracture at any osteoporotic site in Canadians [4, 7–10]. This study of the prospective CaMos cohort (*N* = 7689; 72% women) which was younger (mean age 66 and IQR 59–73 years) than our cohort observed age-adjusted hazard ratios (95% CI) of 1.43 (1.24–1.64) and 1.42 (1.12–1.79) in fractured women and men, respectively [4]. The higher relative mortality risk increase post-fracture observed in our cohort compared to the CaMos cohort was likely partly driven by the higher number of hip fractures in our older cohort, since hip fracture numbers increase in older individuals. The mortality rate observed in our cohort was approximately 2-fold higher in hip fracture compared to non-hip fracture cases in both women and men over the three years post-fracture. However, non-hip fractures also contributed to increased mortality rate observed for all fractures combined, considering the 1.4–1.9- and 1.7–2.4-times greater mortality rate observed, respectively, in women and men with non-hip fracture vs no fracture. Data on non-hip fracture mortality rates in similar cohorts is lacking; however, the 1-year hip fracture mortality rate of 21.5% in women and 32.3% in men observed in our cohort was relatively similar to a 2013 Canadian cohort study of adults aged ≥ 65 which observed a 1-year hip fracture mortality rate of 22% in women and 33% in men [9]. Although not as common as hip fracture, femur, multisite, pelvis and vertebral fractures were also associated with relatively high 1-year mortality rate in this cohort compared to other fracture sites. Prior studies conducted in similar cohorts have also reported significantly increased mortality after pelvis and vertebral fractures [4, 7, 8].

The relative difference in mortality rate between fracture and non-fracture cases was observed to decline with increasing age in both women and men, although it remained approximately 2-3x higher even in women and men aged 86+ years. This age-related trend was also observed in prior research and has been attributed to younger adults having much lower absolute mortality rates and as such the excess mortality discrepancy between the younger and older groups may reflect the life expectancy gap in the general population [33]. Alternatively, younger individuals who experience fragility fracture at younger age may have a poorer health status for their age, compared to older individuals with fragility fractures [33]. The latter explanation calls for urgency to prevent fragility fractures in younger populations, however, further research is needed to help elucidate this age discrepancy.

Gender trends in our study showed that, although men made up about one-quarter of index fracture cases, they contributed to about one-third of deaths. Although the mortality rate was also higher in men vs women in the non-fracture cohort, this gender difference was slightly larger in the fracture cohort for all fractures combined, hip fractures or non-hip fractures. Compared to women, Canadian men were also previously observed to have significantly higher mortality risk after hip [8, 9], vertebral [4, 7, 8], and humerus fractures [4, 8]. Interestingly, the range in absolute mortality rate based on age was found to be wider in women with approximately 6–7-fold difference between the oldest and youngest age groups compared to men with approximately 4–5-fold difference. Perhaps younger men with fragility fracture may be more vulnerable than older men when compared to women of their respective age groups, or alternatively older women approach closer to older men in terms of their vulnerability. Although we observed a higher relative risk difference in mortality rate between women and men of younger age groups compared to older age groups, this observation was not unique to the fracture cohort. Further, research examining mortality rate post-facture and interactions between sex and age is needed to help confirm and elucidate these observations.

Strengths of this study include having examined large fracture and non-fracture cohorts closely matched on age, sex, geography and comorbidities. A hard match method was used due to the limited number of covariates that were deemed important from a clinical point of view, and 88% of the full unmatched cohort was closely matched with the non-fracture cohort on these covariates. The matched fracture cohort was also similar to the full unmatched fracture cohort, supporting the generalizability of the findings of the matched analysis. Real-world data was captured through all fracture care-related public healthcare services from a province contributing to over one-third of fractures nationally [34]. Estimates of mortality risks in Canadian adults were reported as up to date, with previous studies relying on 1986–2013 data [4, 7–10]. Finally, Canadians aged > 65 were examined, who based on age alone are recommended for fracture risk assessments due to their age-related fracture risk [24], but have not been the focus of prior non-hip fracture mortality studies [4, 7–10].

Limitations of this study include the potential for residual confounding which always exists in any observational study, even after yielding a good match in our study based on a number of clinically relevant potential confounders. Another limitation stems from estimating crude mortality risks without adjusting for potential confounders that could not be used during matching, such as a number of and/or time to second fracture, which was previously found to be associated with increased mortality risk [6]. By excluding patients from the fracture cohort who had another fracture 5 years prior to their index event, but not beyond those 5 years, the fracture cohort was potentially biased towards an older population; this resulted in a mean age roughly 5 years higher than expected for adults aged > 65 [35]. By excluding patients from the non-fracture cohort who experienced a fracture 5 years prior to their index date, but not beyond those 5 years, the non-fracture cohort might have included a small proportion of fractured patients. There may be an underestimation of the number of fractures in this cohort, particularly non-hip, considering that only the ‘Most Responsible Diagnosis (ie, if no definite diagnosis was made, the main symptom, abnormal finding, or problem was selected)’ [26] and ‘Pre-Admit Comorbidity’ were used to identify index fractures. Vertebral fractures were likely underestimated considering that two-thirds are typically silent and that ‘Most Responsible Diagnosis’ was used to identify index fractures [36, 37]. Vertebral fractures could also be more thoroughly identified using the Wright et al. [27] algorithm which takes into account imaging codes in addition to fracture diagnosis codes. As in prior healthcare database research, the determination of fragility fracture was based on the exclusion of high-trauma ICD codes and not independent adjudication [9], which may have under/overestimated the number of fragility fractures in this cohort. Cause of death was unavailable and represents an opportunity for further research. Finally, while these results may be generalizable to populations similar to that of Ontario, there may be less generalizability to specific race and ethnic groups [27].

.

## Conclusion

In this large, real-world cohort of Canadian adults aged > 65 years, a fragility fracture at any site was associated with reduced survival up to 6 years post-fracture, with the greatest reduction occurring within the first year, when mortality risk more than doubled. Within the first year, deaths were observed in 1 in 8 women and 1 in 5 men following any fracture, in 1 in 11 women and 1 in 7 men following a non-hip fracture, and in 1 in 5 women and 1 in 3 men following a hip fracture.

## Supplementary Information

**Additional file 1: Supplementary Table 1.** Primary databases used for the study. **Supplementary Table 2.** Diagnosis Codes for Fragility Fractures. **Supplementary Table 3.** Diagnosis Codes for Trauma Fractures. **Supplementary Table 4.** CCI codes for fragility fracture-related surgical technique procedures. **Supplementary Table 5.** Diagnosis codes for fragility fracture-related complications. **Supplementary Table 6.** Clinical characteristics of the full, unmatched fracture cohort. **Supplementary Table 7.** Index fragility fractures by age and calendar year. **Supplementary Figure 1.** Study schema.

## Data Availability

The data that support the findings of this study are available from ICES but restrictions apply to the availability of these data, which were used under license for the current study, and so are not publicly available [https://www.ices.on.ca/Data-and-Privacy/ICES-data] [38]. Data are however available from the authors upon reasonable request and with permission of ICES.

## Abbreviations

CI: Confidence interval
CCI: Canadian Classification of Health Interventions
COPD: Chronic obstructive pulmonary disorder
DAD: Discharge Abstract Database
ICD: International Classification of Diseases
IKN: ICES-specific key number
NACRS: National Ambulatory Care Reporting System
OHIP: Ontario Health Insurance Plan
RPDB: Registered Persons Database
SDS: Same Day Surgery
