# Supplementary material for: Mortality in older adults following a fragility fracture: real-world retrospective matched-cohort study in Ontario
Source: BMC Musculoskelet Disord. 2021 Jan 23;22:105. doi: 10.1186/s12891-021-03960-z (PMC7824940; doi:10.1186/s12891-021-03960-z)
Supplement: Supplementary file 1 — Additional file 1: Supplementary Table 1. Primary databases used for the study. Supplementary Table 2. Diagnosis Codes for Fragility Fractures. Supplementary Table 3. Diagnosis Codes for Trauma Fractures. Supplementary Table 4. CCI codes for fragility fracture-related surgical technique procedures. Supplementary Table 5. Diagnosis codes for fragility fracture-related complications. Supplementary Table 6. Clinical characteristics of the full, unmatched fracture cohort. Supplementary Table 7. Index fragility fractures by age and calendar year. Supplementary Figure 1. Study schema. [file 12891_2021_3960_MOESM1_ESM.docx]

## Supplementary materials

**Supplementary Table 1.** Primary databases used for the study.

**Supplementary Table 2.** Diagnosis Codes for Fragility Fractures

**Supplementary Table 3.** Diagnosis Codes for Trauma Fractures

**Supplementary Table 4.** CCI codes for fragility fracture-related surgical technique procedures

**Supplementary Table 5.** Diagnosis codes for fragility fracture-related complications

**Supplementary Table 6.** Clinical characteristics of the full, unmatched fracture cohort

**Supplementary Table 7.** Index fragility fractures by age and calendar year

**Supplementary Figure 1.** Study schema

Supplementary Table 1. Primary databases used for the study

| **Database** | **Type of care/ professions** |
| --- | --- |
| Registered Persons Database (RPDB) | Demographic information including an individual's date of death |
| Discharge Abstract Database (DAD)/ Same Day Surgery (SDS) | Inpatient hospital discharge summaries |
| National Ambulatory Care Reporting System (NACRS) | Emergency and ambulatory care visits |
| Ontario Health Insurance Plan (OHIP) | Physician billings to the Ontario Health Insurance Plan (OHIP) |

Supplementary Table 2. Diagnosis Codes for Fragility Fractures

| **Fracture type** | **ICD-10 Code** |
| --- | --- |
| Hip | S72.0 - Fracture of neck of femur  S72.1 - Pertrochanteric fracture   - Intertrochanteric fracture - Trochanteric fracture   S72.2 - Subtrochanteric fracture |
| Radius and Ulna | S52.0 - Fracture of upper end of ulna  S52.1 - Fracture of upper end of radius  S52.2 - Fracture of shaft of ulna  S52.3 - Fracture of shaft of radius  S52.4 - Fracture of shafts of both ulna and radius  S52.7 - Multiple fractures of forearm  S52.8 - Fracture of other parts of forearm   - Lower end of ulna - Head of ulna   S52.9 - Fracture of forearm, part unspecified |
| Wrist | S52.5 - Fracture of lower end of radius  S52.6 - Fracture of lower end of both ulna and radius |
| Humerus/ Shoulder | S42.2 - Fracture of upper end of humerus  S42.3 - Fracture of shaft of humerus  S42.4 - Fracture of lower end of humerus  S42.8 - Fracture of other parts of shoulder and upper arm  S42.9 - Fracture of shoulder girdle, part unspecified  Fracture of shoulder NOS |
| Vertebral | S22.0 - Fracture of thoracic vertebra, Fracture of thoracic spine NOS  S22.1 - Multiple fractures of thoracic spine  S32.0 - Fracture of lumbar vertebra, fracture of lumbar spine |
| Femur | S72.3 - Fracture of shaft of femur  S72.4 - Fracture of distal end of femur  S72.7 - Multiple fractures of femur  S72.8 - Fractures of other parts of femur  S72.9 - Fracture of femur, part unspecified   - Applicable To Fracture of thigh NOS Fracture of upper leg NOS - Type 1 exclusion for fracture of hip NOS |
| Tibia, fibula, and knee | S82.0 - Fracture of patella   - Knee cap   S82.1 - Fracture of upper end of tibia   - With or without mention of fracture of fibula   S82.2 - Fracture of shaft of tibia   - With or without mention of fracture of fibula   S82.3 - Fracture of lower end of tibia   - With or without mention of fracture of fibula   S82.4 - Fracture of fibula alone  S82.5 - Fracture of medial malleolus   - Tibia involving   S82.6 - Fracture of lateral malleolus   - Fibula involving |
| Sternum and clavicle | S22.2 – Fracture of sternum  S42.0 - Fracture of clavicle |
| Ribs | S22.3 – Fracture of rib  S22.4 – Multiple fractures of rib |
| Pelvis | S32.1 - Fracture of sacrum  S32.2 - Fracture of coccyx  S32.3 - Fracture of ilium  S32.4 - Fracture of acetabulum  S32.5 - Fracture of pubis  S32.7 - Multiple fractures of lumbar spine and pelvis  S32.8 - Fracture of other and unspecified parts of lumbar spine and pelvis   - Fracture of: - ischium - lumbosacral spine NOS - pelvis NOS |
| Multiple Fracture | S42.7 - Multiple fractures of clavicle, scapula and humerus  T02.1 - Fractures involving thorax with lower back and pelvis  T02.2 - Fractures involving multiple regions of one upper limb  T02.3 - Fractures involving multiple regions of one lower limb  T02.4 - Fractures involving multiple regions of both upper limbs  T02.5 - Fractures involving multiple regions of both lower limbs  T02.6 - Fractures involving multiple regions of upper limb(s) with lower limb(s)  T02.7 - Fractures involving thorax with lower back and pelvis with limb(s)  T02.8 - Fractures involving other combinations of body regions  T02.9  - Multiple fractures, unspecified |

Exclusions: invalid linkage number; event date after the date of death; missing age or sex; older than 105 years; non-resident of Ontario

Supplementary Table 3. Diagnosis Codes for Trauma Fractures*

| **Trauma Code** | **ICD-10 Code** |
| --- | --- |
| Accidents | V01-V99, X00-X58, X59.9, W20-W99 |
| Injuries involving multiple body regions | T00-T01, T03-T07 |
| Falls | W02-W04, W09, W11-W17 |

*Trauma codes above represent causes for high trauma fracture and were excluded due to the study’s purpose of analyzing low trauma fractures.

Supplementary Table 4. CCI codes for fragility fracture-related surgical technique procedures

|  | **Initial Surgeries (request R code status)**  ***Must have no R attribute (inatstat)** | **Revision Surgeries (request R code status)**  **** surgeries listed below are revision surgeries; in addition, any “Initial Surgery” with an R attribute (inatstat) is also considered a revision surgery.** |
| --- | --- | --- |
| Spinal Vertebrae (1.SC) | 1.SC.74 - Fixation  1.SC.75 - Fusion  1.SC.87 - Excision partial  1.SC.89 - Excision total | 1.SC.54 - Management of internal device  1.SC.55 - Removal of device or appliance  1.SC.80 - Repair |
| Intervertebral Disc (1.SE.) | 1.SE.53 - Implantation of internal device  1.SE.87 - Excision partial | 1.SE.55 - Removal of device |
| Sacrum and Coccyx (1.SF.) | 1.SF.73 - Reduction  1.SF.74 - Fixation  1.SF.87 - Excision partial  1.SF.89 - Excision total  1.SF.91 - Excision radical | 1.SF.55 - Removal of device  1.SF.80 - Repair |
| Sacroiliac Joint (1.SI) | 1.SI.74 - Fixation  1.SI.75 - Fusion | none. |
| Sternum (1.SK.) | 1.SK.73 - Reduction  1.SK.74 - Fixation  1.SK.87 - Excision partial | 1.SK.55 - Removal of device  1.SK.80 - Repair |
| Ribs (1.SL.) | 1.SK.58 - Procurement  1.SK.73 - Reduction  1.SK.74 - Fixation  1.SK.87 - Excision partial  1.SK.89 - Excision total  1.SK.91 - Excision radical | 1.SK.54 - Management of internal device  1.SK.55 - Removal of device or appliance  1.SK.79 - Repair by increasing size  1.SK.80 - Repair |
| Clavicle (1.SM.) | 1.SM.73 - Reduction  1.SM.74 - Fixation  1.SM.80 - Repair  1.SM.87 - Excision partial | 1.SM.55 - Removal of device |
| Scapula (1.SN.) | 1.SN.58 - Procurement  1.SN.72 - Release  1.SN.73 - Reduction  1.SN.74 - Fixation  1.SN.75 - Fusion  1.SN.87 - Excision partial  1.SN.91 - Excision radical  1.SN.93 - Amputation | 1.SN.55 - Removal of device |
| Pelvis (1.SQ.) | 1.SQ.53 - Implantation of internal device  1.SQ.58 - Procurement  1.SQ.73 - Reduction  1.SQ.74 - Fixation  1.SQ.83 - Transfer  1.SQ.87 - Excision partial  1.SQ.91 - Excision radical  1.SQ.93 - Amputation | 1.SQ.55 - Removal of device  1.SQ.80 - Repair |
| Pubis (1.SW.) | 1.SW.73 - Reduction  1.SW.74 - Fixation  1.SW.87 - Excision partial | 1.SW.55 - Removal of device |
| Shoulder Joint (1.TA.) | 1.TA.53 - Implantation of internal device  1.TA.58 - Procurement  1.TA.72 - Release  1.TA.73 - Reduction  1.TA.74 - Fixation  1.TA.75 - Fusion  1.TA.83 - Transfer  1.TA.87 - Excision partial  1.TA.93 - Amputation | 1.TA.55 - Removal of device  1.TA.80 - Repair |
| Acromioclavicular and sternoclavicular joints (1.TB.) | 1.TB.72 - Release  1.TB.73 - Reduction  1.TB.74 - Fixation  1.TB.87 - Excision partial | 1.TB.55 - Removal of device  1.TB.80 - Repair |
| Humerus (1.TK.) | 1.TK.58 - Procurement  1.TK.73 - Reduction  1.TK.74 - Fixation  1.TK.82 - Reattachment  1.TK.83 - Transfer  1.TK.87 - Excision partial  1.TK.91 - Excision radical  1.TK.93 - Amputation | 1.TK.55 - Removal of device  1.TK.79 - Repair by increasing size  1.TK.80 - Repair |
| Radius and ulna (1.TV.) | 1.TV.58 - Procurement  1.TV.73 - Reduction  1.TV.74 - Fixation  1.TV.82 - Reattachment  1.TV.83 - Transfer  1.TV.84 - Construction or Reconstruction  1.TV.87 - Excision partial  1.TV.91 - Excision radical  1.TV.93 - Amputation | 1.TV.55 - Removal of device  1.TV.79 - Repair by increasing size  1.TV.80 - Repair |
| Wrist joint (1.UB.) | 1.UB.53 - Implantation of internal device  1.UB.58 - Procurement  1.UB.72 - Release  1.UB.73 - Reduction  1.UB.74 - Fixation  1.UB.75 - Fusion  1.UB.83 - Transfer  1.UB.87 - Excision partial  1.UB.93 - Amputation | 1.UB.55 - Removal of device  1.UB.80 - Repair |
| Hip (1.VA.) | 1.VA.53 - Implantation of internal device  1.VA.58 - Procurement  1.VA.72 - Release  1.VA.73 - Reduction  1.VA.74 - Fixation  1.VA.75 - Fusion  1.VA.83 - Transfer  1.VA.87 - Excision partial  1.VA.93 - Amputation | 1.VA.55 - Removal of device  1.VA.80 - Repair |
| Femur (1.VC.) | 1.VC.58 - Procurement  1.VC.73 - Reduction  1.VC.74 - Fixation  1.VC.82 - Reattachment  1.VC.83 - Transfer  1.VC.87 - Excision partial  1.VC.91 - Excision radical  1.VC.93 - Amputation | 1.VC.55 - Removal of device  1.VC.79 - Repair by increasing size  1.VC.80 - Repair |
| Knee joint (1.VG.) | 1.VG.53 - Implantation of internal device  1.VG.58 - Procurement  1.VG.72 - Release  1.VG.73 - Reduction  1.VG.74 - Fixation  1.VG.80 - Repair  1.VG.83 - Transfer  1.VG.87 - Excision partial  1.VG.93 - Amputation | 1.VG.55 - Removal of device |
| Patella (1.VP.) | 1.VP.53 - Implantation of internal device  1.VP.72 - Release  1.VP.73 - Reduction  1.VP.74 - Fixation  1.VP.87 - Excision partial  1.VP.89 - Excision total | 1.VP.55 - Removal of device  1.VP.80 - Repair |
| Tibia and Fibula (1.VQ.) | 1.VQ.58 - Procurement  1.VQ.73 - Reduction  1.VQ.74 - Fixation  1.VQ.82 - Reattachment  1.VQ.83 - Transfer  1.VQ.87 - Excision partial  1.VQ.91 - Excision radical  1.VQ.93 - Amputation | 1.VQ.55 - Removal of device  1.VQ.79 - Repair by increasing size  1.VQ.80 - Repair |
| Ankle joint (1.WA.) | 1.WA.53 - Implantation of internal device  1.WA.58 - Procurement  1.WA.72 - Release  1.WA.73 - Reduction  1.WA.74 - Fixation  1.WA.75 - Fusion  1.WA.83 - Transfer  1.WA.87 - Excision partial  1.WA.93 - Amputation | 1.WA.55 - Removal of device  1.WA.80 - Repair |

Supplementary Table 5. Diagnosis codes for fragility fracture-related complications

| **Complication** | **ICD-10 codes (DADSDS, NACRS)** | **DXCODE code (OHIP)** |
| --- | --- | --- |
| Infections related to surgery | T81.4 - Infection following a procedure, not elsewhere classified  T84.5 - Infection and inflammatory reaction due to internal joint prosthesis  T84.6 - Infection and inflammatory reaction due to internal fixation device [any site]  T84.7 - Infection and inflammatory reaction due to other internal orthopaedic prosthetic devices, implants and grafts  T85.7 - Infection and inflammatory reaction due to other internal prosthetic devices, implants and grafts | 998 – of surgical and medical care (e.g. wound infection, wound disruption, other iatrogenic disease) |
| Complications related to prosthetic devices | T84 - Complications of internal orthopaedic prosthetic devices, implants and grafts | None. |
| VTE (includes DVT and PE) | I26 - Pulmonary embolism  I80 - Phlebitis and thrombophlebitis  I81 - Portal Vein Thrombosis  I82 - Other venous embolism and thrombosis  I63.6 - Cerebral infarction due to cerebral venous thrombosis, nonpyogenic  I67.6 - Nonpyogenic thrombosis of intracranial venous system | None. |
| Pneumonia | J12 - Viral pneumonia, not elsewhere classified  J13 - Pneumonia due to Streptococcus pneumoniae  J14 - Pneumonia due to Haemophilus influenzae  J15 - Bacterial pneumonia, not elsewhere classified  J16 - Pneumonia due to other infectious organisms, not elsewhere classified  J17 - Pneumonia in diseases classified elsewhere  J18 - Pneumonia, organism unspecified  J22 - Unspecified acute lower respiratory infection  J95 - Postprocedural respiratory disorders, not elsewhere classified | 486 - Pneumonia, all types |
| Myocardial Infarction | I21 - Acute Myocardial Infarction  I22 - Subsequent Myocardial Infarction | None. |
| Fracture resulting from surgery/periprosthetic fracture | M96.6 - Fracture of bone following insertion of orthopaedic implant, joint prosthesis, or bone plate | None. |

Supplementary Table 6. Clinical characteristics of the full, unmatched fracture cohort

| **Clinical Characteristics** | **Full, unmatched fracture cohort n (%)** |
| --- | --- |
| **Total number of patients** | 115,776 |
| **Sex**  Female  Male | 83,690 (72.3%)  32,086 (27.7%) |
| **Age**  Mean ± SD^a^  Median (IQR)^a^  66-70 years  71-75 years  76-80 years  81-85 years  ≥86 years | 80.41 ± 8.28  81 (74-87)  17,998 (15.5%)  17,847 (15.4%)  20,596 (17.8%)  24,119 (20.8%)  35,216 (30.4%) |
| **Urban Residence** | 103,720 (89.6%) |
| **Respiratory conditions^b^**  Asthma  COPD | 17,538 (15.1%)  33,485 (28.9%) |
| **Inflammatory conditions^b^**  Rheumatoid arthritis  Psoriasis  Spondyloarthritis | 4,459 (3.9%)  8,076 (7.0%)  5,084 (4.4%) |
| **Cancer^b^** | 8,390 (7.2%) |
| **Chronic kidney disease^b^** | 13,757 (11.9%) |
| **Diabetes^b^** | 35,434 (30.6%) |
| **Vascular events^b^**  Myocardial infarction  Stroke or cerebrovascular events | 8,175 (7.1%)  35,030 (30.3%) |
| **Osteoarthritis^b^** | 88,223 (76.2%) |
| **Dementia^b^** | 24,092 (20.8%) |
| **Osteoporosis treatment type within 1 year prior^a,c^**  Any treatment  Denosumab  Bisphosphonate  Raloxifene  HRT | 32,757 (28.3%)  1,578 (1.4%)  29,030 (25.1%)  656 (0.6%)  3,597 (3.1%) |
| **Index fracture by site^a,d^**  Hip  Wrist  Clavicle/ribs/sternum  Humerus  Tibia/fibula/knee  Pelvis  Vertebral  Radius/ulna  Multisite  Femur  Any site | 31,613 (27.3%)  17,859 (15.4%)  14,559 (12.6%)  13,237 (11.4%)  10,894 (9.4%)  8,328 (7.2%)  7,721 (6.7%)  4,828 (4.2%)  3,735 (3.2%)  3,002 (2.6%)  115,776 (100%) |
| **Second fracture by site^a,d^**  Hip  Clavicle/ribs/sternum  Wrist  Humerus  Pelvis  Vertebral  Multisite  Tibia/fibula/knee  Radius/ulna  Femur  Any site | 5,745 (5.0%)  2,460 (2.1%)  2,249 (1.9%)  2,088 (1.8%)  1,977 (1.7%)  1,819 (1.6%)  1,518 (1.3%)  1,317 (1.1%)  741 (0.6%)  715 (0.6%)  20,629 (17.8%) |

Values reported as n (%) unless otherwise indicated; percent of total respective cohort.

*p<0.05, **p<0.001 statistical significance between matched fracture cohort and non-fracture cohort

^a^ Variable not used for matching

^b^ Time frame for cancer was 5 years within index data and for all other comorbidities any time prior to index date.

^c^ Within 1 year of index date. Bisphosphonates include alendronate, cyclical etidronate, risedronate, or zoledronic acid. Denosumab is not publicly covered in men and teriparatide in men or women in Ontario.

^d^ Index fragility fracture cases from January 1, 2011 to March 31, 2015. Second fragility fracture cases from the date of index event to March 31, 2017. Reported from highest to lowest number

Supplementary Table 7. Index fragility fractures by age and calendar year

|  | **Year of index fracture** | | | | |
| --- | --- | --- | --- | --- | --- |
|  | **2011** | **2012** | **2013** | **2014** | **2015^a,b^** |
| Length of Follow-up (days) | 2190 | 1825 | 1460 | 1095 | 730 |
| Proportion of the fracture cohort (%)^a^ | 21.7% | 22.5% | 24.2% | 25.4% | 6.2% |

^a^ Proportions are based on the full fracture cohort (N=115,775) which also included patients who were not able to be matched to the control cases.

Note: Follow-up was completed for all patients on March 31, 2017. Patients with an index fragility fracture incurred between January 1, 2011 and March 31, 2015 were included in this cohort.

Supplementary Figure 1. Study schema

Index event identification period

(Jan 1, 2011 to Mar 31, 2015)

5-Year lookback period

(Jan 1, 2006 to Jan 1, 2011)

Data collection period

(2 years to up to 6 years)

Maximum follow-up date

(Mar 31, 2017)

INDEX EVENT DATE

Note: Index event definition include fracture occurring at a fragility fracture site, excluding non-osteoporotic fracture sites (ie, skull, face, hands, and feet) or fractures associated with a trauma code

Exclusions: invalid linkage number; event date after the date of death; missing age or sex; older than 105 years; non-resident of Ontario
